# Hypotensive Snake Venom Components—A Mini-Review

**DOI:** 10.3390/molecules24152778

**Published:** 2019-07-31

**Authors:** Orsolya Péterfi, Francisc Boda, Zoltán Szabó, Elek Ferencz, László Bába

**Affiliations:** 1 Faculty of Pharmacy, University of Medicine, Pharmacy, Science and Technology of Targu Mures, Gheorghe Marinescu Street No. 38, 540139 Tirgu Mures, Romania; 2 Department of Fundamental Pharmaceutical Sciences, Faculty of Pharmacy, University of Medicine, Pharmacy, Science and Technology of Targu Mures, Gheorghe Marinescu Street No. 38, 540139 Tirgu Mures, Romania; 3 Department of Specialty Pharmaceutical Sciences, Faculty of Pharmacy, University of Medicine, Pharmacy, Science and Technology of Targu Mures, Gheorghe Marinescu Street No. 38, 540139 Tirgu Mures, Romania

**Keywords:** snake venom, hypotensive peptides, bradykinin potentiating peptides, natriuretic peptides, phospholipases A_2_

## Abstract

Hypertension is considered a major public health issue due to its high prevalence and subsequent risk of cardiovascular and kidney diseases. Thus, the search for new antihypertensive compounds remains of great interest. Snake venoms provide an abundant source of lead molecules that affect the cardiovascular system, which makes them prominent from a pharmaceutical perspective. Such snake venom components include bradykinin potentiating peptides (proline-rich oligopeptides), natriuretic peptides, phospholipases A_2_, serine-proteases and vascular endothelial growth factors. Some heparin binding hypotensive factors, three-finger toxins and 5′ nucleotidases can also exert blood pressure lowering activity. Great advances have been made during the last decade regarding the understanding of the mechanism of action of these hypotensive proteins. Bradykinin potentiating peptides exert their action primarily by inhibiting the angiotensin-converting enzyme and increasing the effect of endogenous bradykinin. Snake venom phospholipases A_2_ are capable of reducing blood pressure through the production of arachidonic acid, a precursor of cyclooxygenase metabolites (prostaglandins or prostacyclin). Other snake venom proteins mimic the effects of endogenous kallikrein, natriuretic peptides or vascular endothelial growth factors. The aim of this work was to review the current state of knowledge regarding snake venom components with potential antihypertensive activity and their mechanisms of action.

## 1. Introduction

Cardiovascular diseases are the leading cause of death worldwide as they account for nearly 18 million deaths yearly. The pathophysiological risk factors for heart diseases include elevated blood pressure, diabetes, hyperlipidaemia, obesity and high blood glucose levels, while the main behavioral risk factors are unhealthy diet, lack of physical activity, smoking and alcohol consumption [1,2].

Hypertension (HT) is involved in approximately 45% of cases of heart disease-related deaths, which makes it a major public health issue [2]. In 2010 the prevalence of high blood pressure was 31% among adults worldwide which represents a 5.2% increase in global prevalence since 2000 [3]. It is estimated that the incidence of hypertension in Europe will increase by 15–20% by 2025 [4].

Primary HT tends to develop gradually without an identifiable cause, while secondary HT can be attributed to conditions, such as primary hyperaldosteronism, Cushing’s syndrome, pheochromocytoma, renovascular hypertension or sleep apnea. Secondary HT can also appear as a side effect of different drugs [5,6]. Risk factors for the development of HT are high salt consumption, lack of exercise, smoking, excessive alcohol consumption, advanced age and high stress levels [7]. Decreasing the blood pressure by lifestyle changes and use of drug therapy can reduce premature morbidity and mortality caused by HT. Studies show that lowering the systolic blood pressure to 130 mmHg decreases the risk of major cardiovascular events, coronary heart disease, stroke, heart failure, renal failure, and all-cause mortality [8,9].

An outstanding advancement in cardiovascular pharmacology was the discovery of angiotensin-converting enzyme (ACE) inhibitors. Captopril, the first ACE inhibitor approved for human use was developed based on the structure of a bradykinin potentiating peptide isolated from the venom of the Brazilian pit viper, *Bothrops jararaca* [10]. The development of captopril shows the potential of snake venom components to function as lead molecules in modern drug development. Thus, snake venoms offer a vast number of possible lead molecules due to the various biochemical and pharmacological activities possessed by their components. Putative applications include antihypertensive, anticoagulant, antimitotic and antibacterial therapies, pain management and treatment of multiple sclerosis or other neurological disorders [11].

Venomous animals use various envenomation strategies to immobilize, kill and commence the digestion of prey. Biochemical mechanisms involved in the immobilization of prey are paralysis and/or hypotension, which limit prey flight and resistance through direct or indirect/synergistic biochemical processes. In addition to circulatory shock and rapid prey immobilization, hypotension may contribute to the diffusion of other snake venom components [12,13]. Pulmonary vascular obstruction and coronary ischemia caused by snake venoms can further lead to decrease in blood pressure [14]. Proteins and peptides with direct hypotensive activity exert their action by binding to specific endogenous molecular targets (receptors, enzymes, channels). This makes them significant from a pharmaceutical point of view [15].

Snake venoms are complex mixtures of proteins, peptides and other small organic and inorganic molecules, all having a synergistic effect [16,17]. Venom composition varies between species and is further influenced by age, diet, habitat, sexual dimorphism and seasonal variations [18,19]. Snake venoms reach the physiological systems of the prey via its bloodstream, leading to neurological and/or cardiovascular disorders [20], such as blood pressure variation, erythrocyte destruction, local and systemic hemorrhages, arrhythmia, tachycardia, muscle paralysis and possible cardiac arrest [15,21,22].

The aim of this work was to review the current state of knowledge regarding snake venom components with potential antihypertensive activity and their mechanisms of action.

## 2. Overview of Hypotensive Mechanisms

Angiotensin-converting enzyme (ACE) increases blood pressure by increasing the concentration of angiotensin (AT) II. In the same time, it transforms bradykinin (BK) into an inactive metabolite BK-(1-7). The stimulation of AT II receptor type 1 leads to vasoconstriction of the systemic arterioles, Na^+^ reabsorption and water retention, partially by increasing the secretion of aldosterone from the adrenal gland [23,24]. The inhibition of BK degradation by bradykinin potentiating peptides (BPPs) and the BK synthesis by kallikrein-like snake venom serine-proteases (SVSPs) increase the BK concentration. As a result, bradykinin receptor B_2_ (B_2_-R) stimulation induces vasodilation, anti-fibrosis, anti-inflammatory effect and anti-reactive oxygen species through various intracellular mechanisms. BPPs such as *Bj*-PRO-7a and *Bj*-PRO-13a can bind to muscarinic M_1_/M_3_ receptors (M_1_/M_3_-Rs) as well [25,26].

B_2_-Rs, M_1_/M_3_-Rs and vascular endothelial growth factor receptors (VEGFRs) activate the phospholipase C (PLC) enzyme, which catalyzes the hydrolysis of phosphatidylinositol (PIP_2_) and generates the second messengers inositol trisphosphate (IP_3_) and diacylglycerol (DAG). Stimulation of the IP_3_ receptor in the endoplasmic reticulum membrane leads to the increase of intracellular Ca^2+^ concentration [27]. Elevated Ca^2+^ levels lead to the activation of two distinct pathways.

Through the first pathway, protein kinase B (Akt) and Ca^2+^/calmodulin-dependent protein kinase (CaM-K) induce the phosphorylation of endothelial nitric synthase (eNOS), which synthesizes nitric oxide (NO) and l-citrulline from l-arginine. l-citrulline is reconverted into l-arginine via the argininosuccinate pathway catalyzed by the argininosuccinate synthase (AsS) [28,29]. NO binds to soluble guanylate cyclase (sGC) in the smooth/cardiac muscle cell, which can also be activated by snake venom natriuretic peptides (SVNPs) in the myocardium. sGC catalyzes the conversion of guanosine triphosphate (GTP) to cyclic guanosine monophosphate (cGMP), that regulates cellular activity through protein kinases. Protein kinase G (PKG) stimulates myosin light chain phosphatase (MLCP) activity and P-type calcium channels (PTCCs) while inhibiting l-type calcium channels (LTCCs), thus leading to vasorelaxation. LTCC function can further be inhibited by LTCC blockers [30,31].

The second pathway involves phospholipase A_2_ (PLA_2_) activation. Arachidonic acid is produced through membrane phospholipid hydrolysis and it is metabolized by cyclooxygenase-2 (COX-2) to produce prostaglandin I_2_ (PGI_2_). Stimulation of prostacyclin receptor (IP) by PGI_2_ and binding of adenosine to adenosine A_2_ receptor (A_2_-R) increases intracellular cyclic adenosine monophosphate (cAMP) concentration causing protein kinase A (PKA) phosphorylation and inhibition of myosin light chain kinase (MLCK) activity [32,33].

**Figure 1:**
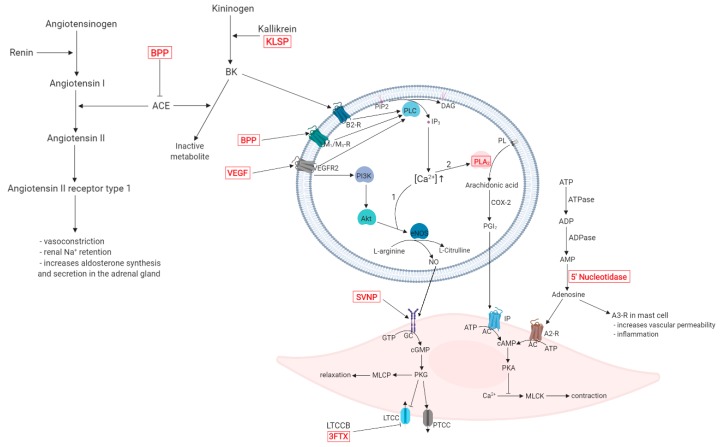
Mechanisms of the hypotensive effect of snake venom components (highlighted in red). Abbreviations: A2-R, A3-R—adenosine A_2_, A_3_ receptors; AC—adenylyl cyclase; ACE—angiotensin-converting enzyme; ADP—adenosine diphosphate; Akt—protein kinase B; AMP—adenosine monophosphate; ATP—adenosine triphosphate; B2-R—bradykinin receptor B_2_; BK—bradykinin; BPP—bradykinin potentiating peptide; cAMP—cyclic adenosine monophosphate; cGMP—cyclic guanosine monophosphate; COX-2—cyclooxygenase-2; DAG—diacylglycerol; eNOS—endothelial nitric synthase; GC—guanylate cyclase; GTP—guanosine triphosphate; IP—prostacyclin receptor; IP_3_—inositol trisphosphate; KLSP—kallikrein-like snake protein; LTCC—L-type calcium channel; LTCCB—L-type calcium channel blocker; M_1_/M_3_-R—muscarinic M_1_/M_3_ receptors; MLCK—myosin light chain kinase; MLCP—myosin light chain phosphate; NO—nitric oxide; PGI_2_—prostaglandin I_2_; PI3K—phosphoinositide 3-kinase; PIP2—phosphatidylinositol; PKA—protein kinase A; PKG—protein kinase G; PL—phospholipid; PLA_2_—phospholipase A_2_; PLC—phospholipase C; PTCC—P-type calcium channel; SVNP—snake venom natriuretic peptide; VEGF—vascular endothelial growth factor; VEGFR2—vascular endothelial growth factor receptor type 2.

Snake venom components are capable of influencing the regulation of blood pressure through their interaction with various enzymes and substrates involved in this complex physiological process. A graphical representation of the described hypotensive mechanisms, including relevant snake venom components and their targets is presented in Figure 1.

## 3. Snake Venom Components with Hypotensive Effects

Experiments concerning snake venoms have described a staggering number of proteins that cause a decrease in blood pressure. These have various biological actions and act through different endogenous systems. Considering the current research, to the extent of this review, we have assigned the hypotensive components to six different classes. The largest class of components consists of those that modulate the kinin system, such as kininogens, activating enzymes and vasoactive products. Another class consists of components having natriuretic peptide-like effects. A third class includes phospholipases that generate highly active mediators through the cyclooxygenase pathway. A fourth class comprises snake venom serine-proteases, having various mechanisms of action. The components of the fifth class strongly resemble the endogenous vascular endothelial growth factors, having similar effects. Last but not least, there is a heterogeneous class tagged as ‘other hypotensive components’ that have less in common compared to the previous classes, but nevertheless are of great importance from our perspective. In the following, we will describe in detail these classes. Individual snake venom components with hypotensive effects are summarized in Table 1.

(i) Bradykinin potentiating peptides (BPP) are proline-rich oligopeptides (PRO) first extracted from the venom of *Bothrops jararaca* [66], which inhibit the ACE and increase the hypotensive capability of bradykinin. ACE converts AT I to AT II, the latter being a vasoconstrictor hormone that indirectly increases blood pressure. Furthermore, ACE leads to the degradation of the vasodilator peptide bradykinin [67]. Bradykinin interacts with G-protein coupled B_2_ receptors, exerts its action through PLC, PLA_2_, prostaglandins and protein kinases and causes changes in the intracellular Ca^2+^ concentration [68]. Stimulation of bradykinin receptor B_2_ leads to the release of NO and prostacyclin, all of which promote vasodilation [69]. In addition to the ACE and BK pathways, BPPs have been shown to possess ACE-independent hypotension inducing mechanisms such as AsS activation, as well as gamma-aminobutyric acid (GABA) and glutamate release in the central nervous system (CNS) [38,70]. AsS catalyzes the l-arginine synthesis from citrulline, necessary for sustaining NO production [71]. BPPs have been identified in numerous viperid and elapid venoms. During the last decade, the hypotensive effect and the mechanism of action of BPPs has been investigated in the case of *Agkistrodon* [44], *Bitis* [41], *Bothrops* [34], *Crotalus* [42] and *Lachesis* [43] species.

(ii) Natriuretic peptides (NPs) are vasoactive hormones that act by two major mechanisms: vasodilation and regulation of renal blood flow. The observed effects include the inhibition of the renin-angiotensin and aldosterone system, diuresis, natriuresis, regulation of vascular permeability and increase of venous capacitance [72,73,74]. Members of the human NP family include the atrial natriuretic peptide (ANP), brain natriuretic peptide (BNP), and C-type natriuretic peptide (CNP). ANP stimulates LTCCs and inhibits T-type calcium currents in the adrenal glomerular cells, inhibits Na^+^ channel in the apical membrane, and also the basolateral Na^+^-K^+^-ATPase in renal tubules [73,75]. BNP and CNP attenuate LTCC currents [76,77]. ANP and BNP bind to A type natriuretic peptide receptors (NPRA), which generate cGMP as an intracellular messenger. Both peptides play an important role in water excretion, cellular growth, electrolyte homeostasis and vascular permeability [78]. Contrarily, CNP binds to B type natriuretic peptide receptors (NPRB), lacks significant natriuretic effect and primarily stimulates bone growth and vascular tone in peripheral veins. Furthermore, all three NP types act on C type natriuretic peptide receptors (NPR-C), which are not linked to cGMP pathways [79,80]. In fact, NPR-C has no intracellular domain, and thus serves as a means of NP clearance, since all three NPs bind to it and following the endocytosis of these, lysosomal degradation occurs [73].

Dendroaspis natriuretic peptide (DNP) is a NP isolated from *Dendroaspis angusticeps* structurally similar to ANP and CNP [81,82]. DNP reduced action potential duration in rabbit ventricular myocytes by activating protein kinase G (PKG), which phosphorylates the α_1c_ subunit of cardiac LTCC proteins [47]. Cenderitide (formerly CD-NP) is a chimeric peptide composed of CNP and the 15 residue C-terminal tail of DNP designed in 2008 to eliminate the unwanted hypotensive properties of NPs while preserving the natriuretic and diuretic effect through the activation of particulate guanylyl cyclase A (pGC-A) and pGC-B [83,84]. Currently the molecule is in clinical trials for heart failure and it can potentially prevent and/or reverse myocardial remodeling [85,86,87].

NP2_Casca from *Crotalus durissus cascavella* venom has a relaxant effect on endothelium-dependent thoracic aortic rings due to a possible involvement of potassium channels. A decrease in heart rate, arterial pressure and an increase in urinary flow, glomerular filtration rate, sodium excretion and nitrite production was observed [48]. Coa_NP is a natriuretic peptide identified in *Crotalus oreganus abyssus* venom that produced endothelium-dependent vasorelaxation in thoracic aortic rings without binding to the guanylate cyclase-coupled NPR-A. Furthermore, the peptide directly and/or indirectly increased NO production [45]. ACE inhibition was observed in the case of PaNP-c *Pseudechis australis* natriuretic peptide and PtNP-a isolated from *Pseudonaja textilis* venom. The latter also exerts similar action to endogenous ANP, increasing intracellular cGMP concentration [50].

Other snake venom NPs have been identified from *Bungarus flaviceps* (KNP) [88,89], *Bungarus multicinctus* [90], *Crotalus durissus cascavella* (NP2_Casca) [48], *Lachesis muta* (Lm-CNP) [91] and *Trimeresurus flavoviridis* (TNP-a, TNPb, TNP-c) [92] venom.

(iii) Phospholipases A_2_ (PLA_2_) hydrolyze the sn-2 acyl bond of glycerophospholipids and contribute to the release of free fatty acids (by far the most important being arachidonic acid) and lysophospholipids. Thus, PLA_2_s mediate prostaglandin and leukotriene synthesis, therefore inflammatory responses through the cascade of arachidonic acid (through cyclooxygenase (COX) and lipoxygenase (LOX) pathways respectively). There are no less than 15 distinct PLA_2_s groups, clustered in four major enzyme types. These are cytosolic PLA_2_s (cPLA_2_), Ca^2+^ independent PLA_2_s (iPLA_2_), secreted PLA_2_s (sPLA_2_) and platelet-activating factor acetylhydrolases (PAF-AH) [93]. Snake venom phospholipases belong to the sPLA_2_ family, exhibiting various effects, including neurotoxicity and cardiotoxicity, inhibition of blood coagulation, induction of oedema and/or interference with platelet function. These proteins are capable of reducing blood pressure through the production of arachidonic acid, a precursor of cyclooxygenase metabolites (prostaglandins or prostacyclin) and interaction with platelets and leukocytes [13,53]. Snake venom PLA_2_s are present in the venoms of species from the Colubridae, Elapidae and Viperidae snake families and they are one of the major toxic components with a wide spectrum of pharmacological effects [94]. Hypotensive PLA_2_s have been identified in the venom of the Elapinae and Viperinae subfamilies. As an example, the peptide OSC3, isolated from *Oxyuranus scutellatus* decreases arterial pressure with the involvement of cyclooxygenase metabolites (prostaglandins or prostacyclin), H_1_-receptors and possibly bradykinin [53].

(iv) Snake venom serine-proteases (SVSP) are trypsin-like enzymes that exert their action on the coagulation cascade, the fibrinolytic and kallikrein–kinin systems and on cells, causing an imbalance in the haemostatic system. SVSPs can imitate the effects of thrombin through the conversion of fibrinogen into fibrin, and the activation of factor V and protein C. Some SVSPs exert direct platelet-aggregating activity by activating plasminogen or coagulation factor XIII [95,96]. A different subgroup of SVMPs, the kallikrein-like SVSPs lower blood pressure by releasing bradykinin from kininogen [59]. Besides their kallikrein-like activity, Tm-VIG and Tm-IIG from *Trimeresurus mucrosquamatus* venom and harobin from *Lapemis hardwickii* venom are shown to reduce blood pressure by degrading angiotensin I to angiotensin II and the latter to two inactive peptides [55,59]. Synergistic action of kallikrein-like SVSPs and BPP has been suggested as they increase BK concentration through different pathways [56,97]. BPPs, NPs and SVSPs are characteristic for neurotoxic Viperidae venoms indicating possible coevolution of hypotensive and paralyzing snake venom strategies [98].

(v) Vascular endothelial growth factors (VEGFs) also known as vascular permeability factors regulate the formation and permeability of blood vessels and maintain the homeostasis through their interaction with kinase-linked receptors. Dimerization of VEGF receptor 1 (VEGFR1) and VEGFR2 induces angiogenesis, while VEGFR3 is responsible for lymphangiogenesis [99,100]. VEGFs induce endothelium-dependent vasorelaxation through the release of NO and PGI_2_ [101]. VEGF-like snake venom proteins have similar biological activities to human VEGF_165_, acting on VEGFR-1 and VEGFR-2, regulating vascular permeability, angiogenesis, mesenchymal cell differentiation and reducing blood pressure [102,103,104].

(vi) Other hypotensive components identified from snake venoms include heparin binding hypotensive factors, three-finger toxins and 5′ nucleotidases.

Heparin is used as an anticoagulant, which exerts its action by binding to antithrombin III and inhibiting clotting enzymes such as thrombin and factor Xa [105]. In addition to its anticoagulant effect, heparin promotes hypotension and vasorelaxation through the activation of endothelial muscarinic M_3_ receptors [106]. A VEGF-like, hypotensive, heparin-binding protein has been identified in *Vipera aspis* venom [64].

Three-finger toxins (3FTX) are a non-enzymatic polypeptide family named after their characteristic spatial structure, in which the three β-stranded loops are cross-linked by four conserved disulphide bridges [107]. 3FTXs are characteristic components in the venoms of elapid snakes, such as *Dendroaspis polylepis* and *Dendroaspis angusticeps*, however they have been identified in the venoms of other snake families as well. 3FTXs have diverse biological functions by interacting with a broad range of receptors [108]. The hypotensive effect of 3FTXs can be attributed to the blockage of LTCC channels and interaction with adrenergic and muscarinic receptors.

LTCC antagonists, also known as LTCC blockers prevent Ca^2+^ ions from entering the cells, which leads to various physiological or pathophysiological responses, depending on the location of the calcium channels. LTCCs are responsible for generating calcium influx in the skeletal, smooth and heart muscles, retina, immune and endocrine cells, thus contributing to gene expression, neurotransmission, cardiac action potential and vasoconstriction [109,110,111]. 3FTXs isolated from snake venoms, such as FS2 and calciseptine act as LTCC antagonists and have a pronounced hypotensive effect [63,112].

5′ nucleotidases are hydrolytic enzymes that play a significant role in prey envenomation. 5′-Nucleotidases interact with factor IX of the blood coagulation cascade and inhibit platelet aggregation. They further act upon adenosine monophosphate (AMP) molecules in order to release adenosine [113], a mechanism through which *Bothrops asper* venom exerts its hypotensive activity [65]. Adenosine is generated by the massive release of ATP from myotubes, and subsequent catalytic decomposition of ATP to ADP, AMP and adenosine by ATPases, ADPases and nucleotidases [65]. The activation of A2-R leads to vasodilation, while mast cell A3-Rs promote vascular permeability and inflammation [114].

## 4. Snake Venom Components as Lead Molecules in Drug Discovery

Snake venom components may be used in their native form, either isolated from venoms or obtained through biological (heterologous expression) or chemical synthesis. Most often, however, the pharmacophore group of the toxins is identified, then used in the synthesis of new therapeutic agents, as in the case of captopril [16,115].

The evolutionary process has led to an increased specificity and selectivity of snake venoms components. Natural toxins are capable of acting selectively on different targets, thus exhibiting diverse biological effects [16,116]. As the toxins only have a limited number of target structures, they can be used as lead molecules for the development and optimization of new active substances [16]. The major disadvantage of natural toxins is their inadequate pharmacokinetic profile. Proteinaceous substances have a low oral bioavailability and require parenteral administration. The short half-life, the susceptibility to proteolytic enzymes and the risk of triggering severe allergic reactions are all factors that limit the use of natural toxins as therapeutic agents [16,117].

BPPs have been the first snake venom components to be used in drug discovery. Structure analysis of BPPs from the venom of *Bothrops jararaca* has highlighted a common amino acid sequence (Phe-Ala-Pro) that functions as a pharmacophore group [118,119]. This sequence has first been modified to allow oral administration, then to increase the binding capability to the active site of ACE [118,120]. The obtained compound, known today as captopril, was the first active substance to be approved for human use that was developed based on a lead molecule from a snake venom [118].

SVNPs exhibit structural similarities with human NPs, but possess greater stability and a more pronounced activity. SVNPs are promising lead molecules for drug development due to their marked hypotensive activity and their resistance against the effect of renal endopeptidases, enzymes responsible for degrading endogenous NPs [84]. Development of synthetic NPs involves the production of chimeric peptides, such as cenderitide (CD-NP). The CD-NP molecule was obtained by fusing the CNP peptide (22 amino acids) with the C-terminal end of DNP (15 amino acids) [83]. The first in vivo studies showed that CD-NP has a diuretic and natriuretic effect, increases glomerular filtration rate, lowers cardiac output and inhibits the renin-angiotensin-aldosterone system [83,87]. Based on these initial results, CD-NP has been proposed for the treatment of chronic heart failure and clinical studies have confirmed its efficacy and favorable safety profile [86,121,122].

Considering the large number of snake venom components, there are vast possibilities to identify and explore lead molecules and pharmacophore groups, which could be used in the development of new active substances for the treatment of hypertension and related cardiovascular diseases. Furthermore, snake venom components are used as molecular tools in biomedical studies that involve intracellular Ca^2+^ concentration and LTCC regulation [123,124].

## 5. Conclusions

With the increasing prevalence of HT worldwide, the search for new blood pressure lowering compounds remains of great interest. Snake venoms provide various hypotensive agents for future diagnostic and pharmacological development. The target specificity and mechanism of action of these snake venom proteins contribute to a more profound understanding of different endogenous systems that regulate blood pressure. This in turn, is of foremost importance in drug development. The current study illustrates that considerable progress has been made in exploring the different hypotensive snake venom components. Even though a considerable number of compounds have already been characterized, further studies are required to determine the various effects and mechanisms of other members of these protein families.

## Figures and Tables

**Table 1:** Snake venom proteins and peptides expressing hypotensive effects through various mechanisms of action.

Protein/Peptide	Source	Mechanism of Hypotensive Effect	Ref.
Bradykinin Potentiating Peptides (BPP)/Proline-Rich Oligopeptides (PRO) *			
*Bj*-PRO-5a	*Bothrops jararaca*	Increases urinary flow rate and sodium excretion. Vasodilation is achieved through the inhibition of the angiotensin-converting enzyme (ACE), along with the activation of bradykinin B_2_ and muscarinic M_1_ receptors. Lowers cardiac output by inducing bradycardia.	[34,35]
*Bj*-PRO-7a	*Bothrops jararaca*	Acts as an M_1_ muscarinic receptor agonist, thus mobilizes intracellular Ca^2+^ in various cell types and induces vasodilation through vascular endothelial cells.	[36]
*Bj*-PRO-9a	*Bothrops jararaca*	Inhibits ACE and increases the effect of endogenous bradykinin (BK).	[37]
*Bj*-PRO-10c (*Bj*-BPP-10c)	*Bothrops jararaca*	Activates argininosuccinate synthetase (AsS) at kidney level, increasing l-arginine and consecutive nitric oxide (NO) production. Increases production of NO through gene expression of AsS and nitric oxide synthase (NOS) in the endothelium. Improves baroreflex sensitivity in the central nervous system and increases the release of gamma-aminobutyric acid (GABA) and glutamate mediators, involved in the regulation of the autonomic nervous system. Lowers cardiac output by inducing bradycardia. Increases urinary flow rate and sodium excretion. Inhibits the angiotensin-converting enzyme (ACE).	[34,38,39,40]
*Bj*-PRO-11e *Bj*-PRO-12b	*Bothrops jararaca*	Lowers cardiac output by inducing bradycardia. Induces Ca^2+^ mobilization in different tissues, possibly interacting with regulators of the cardiovascular system, such as the Ca^2+^/calmodulin-dependent kinase II (CaMK-II). Increases the effect of endogenous BK.	[37]
*Bj*-PRO-13a	*Bothrops jararaca*	Increases AsS activity, NO production and Ca^2+^ mobilization. Agonist on M3 muscarinic receptors (mAChR), possibly inducing smooth muscle relaxation and negative cardiac chronotropy.	[37]
*Bn*-PRO-10a, *Bn*-PRO-10a-MK, *Bn*-PRO-10b-MK, *Br*-PRO-10a, *Bg*-PRO-11a, *Bn*-PRO-10c	*Bitis* spp.	Inhibits the enzymatic activity of ACE with some minor differences regarding modulation of angiotensin I conversion and BK degradation.	[41]
BPP-Cdc	*Crotalus durissus cascavella*	Inhibits BK degradation and conversion of AT I into AT II by inhibiting the enzymatic activity of ACE	[42]
LmrBPP9	*Lachesis muta rhombeata*	Inhibits the enzymatic activity of ACE.	[43]
PRO synthetic analogues	*Agkistrodon bilineatus*	Inhibits the enzymatic activity of ACE.	[44]
**Natriuretic Peptides (NP)**			
Coa_NP	*Crotalus oreganus abyssus*	Induces endothelium dependent vasodilatation through NO formation.	[45,46]
DNP	*Dendroaspis angusticeps*	Phosphorylates Ca^2+^ channel proteins via protein kinase G (PKG) activation, inhibiting L-type Ca^2+^ channel activity in the hearth, modulating contractility.	[47]
NP2_Casca	*Crotalus durissus cascavella*	Increases NO production and consequent vasodilation, also causes increase in urinary flow, glomerular filtration rate and sodium excretion, thus resulting a strong diuretic effect.	[48]
PNP	*Pseudocerastes persicus*	Increases urine flow and sodium excretion thus decreasing blood pressure. It exerts ANP-like activity as it induces cGMP activity and binds to natriuretic peptide receptor (NPR)-A.	[49]
PtNP-a	*Pseudonaja textilis*	Increases intracellular cGMP levels similarly to ANP and BNP while inhibiting ACE activity.	[50]
**Phospholipases A_2_ (PLA_2_)**			
BmooPLA2-I	*Bothrops moojeni*	Decreases blood pressure through an unreported mechanism.	[51]
BthA-I-PLA2	*Bothrops jararacussu*	Decreases blood pressure due to its phospholipase activity.	[52]
OSC3a, OSC3b	*Oxyuranus scutellatus*	Hypotensive effect induced through cyclooxygenase metabolites (dilator prostaglandins or prostacyclin). Possible involvement in the release of endogenous mediators, such as histamine and bradykinin. Both direct (OSC3a) and endothelium-dependent (OSC3a, OSC3b) vasodilator effect.	[53]
**Snake Venom Serine-Proteases (SVSP)**			
AHP-Ka	*Agkistrodon halys pallas*	Possible hypotensive effect, due to its kallikrein-like activity.	[54]
Harobin	*Lapemis hardwickii*	Degrades angiotensin I to angiotensin II and angiotensin II to tetrapeptides lacking hypertensive activity. Releases BK with vasodilator effect, due to its kallikrein-like activity. Decreases blood fibrinogen levels, altering the blood rheology.	[55]
Kn-Ba	*Bitis arietans*	Releases BK and Met-Lys-bradykinin from kininogen, the latter has equivalent biological activity with BK at B1 and B2 receptors causing vasodilation.	[56]
LV-Ka	*Lachesis muta*	Decreases blood pressure through its kallikrein-like activity.	[57]
Rhinocerase	*Bitis gabonica rhinoceros*	Possible hypotensive effect, due to its kallikrein-like activity.	[58]
Tm-VIG and Tm-IIG	*Trimeresurus mucrosquamatus*	Degrades angiotensin I and releases bradykinin from plasma kininogen with potent vasodilator effect.	[59]
**Vascular endothelial Growth Factor Like (VEGF-like) Peptides**			
TfsvVEGF	*Trimeresurus flavoviridis*	Possible hypotensive effect, due to VEGF-like mechanism of action.	[60]
VEGF-F (VR-1’)	*Daboia russelli siamensis*	Possible hypotensive effect, due to VEGF-like mechanism of action.	[61]
**Other Hypotensive Snake Venom Components**			
Calciseptine FS-2 toxin	*Dendroaspis polylepis*	Act as l-Type Ca^2+^ channel blockers.	[62,63]
Heparin-binding dimeric hypotensive factor (HF)	*Vipera aspis*	Exhibits potent hypotensive effect, due to VEGF-like mechanism of action (vasodilation and hyperpermeability).	[64]
Nucleotidases	*Bothrops asper*	Degrades adenosine triphosphate (ATP) to adenosine, which exerts hypotensive activity through vasodilation.	[65]

* While “Bradykinin potentiating peptides” is the well-established name for these proteins, recent publications use the “Proline-rich oligopeptides” expression (based on structure, rather than effect). This is due the fact, that not all known proteins in this class have a bradykinin potentiating effect.
